# The Endocrine System and Alcohol Drinking in Females

**DOI:** 10.35946/arcr.v40.2.02

**Published:** 2020-07-23

**Authors:** Deborah A. Finn

**Affiliations:** 1 Oregon Health & Science University, Portland, Oregon; 2 Veterans Affairs Portland Health Care System, Portland, Oregon

**Keywords:** estrogen, ethanol, glucocorticoid, neurosteroid, progesterone, stress

## Abstract

Sexually dimorphic effects of alcohol exposure throughout life have been documented in clinical and preclinical studies. In the past, rates of alcohol use disorder (AUD) were higher in men than in women, but over the past 10 years, the difference between sexes in prevalence of AUD and binge drinking has narrowed. Recent evidence adds to historical data regarding the influence of sex steroids on alcohol drinking and the interaction with stress-related steroids. This review considers the contribution of the endocrine system to alcohol drinking in females, with a focus on the hypothalamic pituitary gonadal axis and the hypothalamic pituitary adrenal axis and their reciprocal interactions. Emphasis is given to preclinical studies that examined genomic and rapid membrane effects of estrogen, progesterone, glucocorticoids, and GABAergic neurosteroids for their effects on alcohol drinking and models of relapse. Pertinent comparisons to data in males highlight divergent effects of sex and stress steroids on alcohol drinking and emphasize the importance of considering sex in the development of novel pharmacotherapeutic targets for the treatment of AUD. For instance, pharmacological strategies targeting the corticotropin releasing factor and glucocorticoid receptor systems may be differentially effective in males and females, whereas strategies to enhance GABAergic neurosteroids may represent a biomarker of treatment efficacy in both sexes.

## INTRODUCTION

Alcohol use disorder (AUD), a diagnosis that combines criteria for alcohol abuse and alcohol dependence from the 4th edition of the Diagnostic and Statistical Manual of Mental Disorders into a single disorder in the 5th edition,^1^ negatively influences health and is the third-leading preventable cause of death in the United States.^2^ According to the 2015 National Survey on Drug Use and Health, the prevalence of binge drinking, which is the consumption of an excessive amount of alcohol in a short period of time, and of heavy alcohol use was similar in males and females.^2^ Likewise, a recent meta-analysis confirmed a greater increase in alcohol use and binge drinking in women versus men over the past 16 years,^3^ representing a narrowing of the historically higher AUD rate in males. It has been suggested that the increased rate of AUD among women may be due to stress or to drinking to regulate a negative affect.^4^–^6^

As elegantly reviewed by Rachdaoui and Sarkar, acute and chronic alcohol administration disrupts functioning of the endocrine system, which is a complex system of glands that work in conjunction with the nervous system to maintain homeostasis.^7^ Glands of the endocrine system produce and secrete hormones into the circulation, which can have long-lasting as well as rapid actions. Hormones affect physiological functions such as metabolism, reproduction, growth, and development, and they facilitate the ability to respond to changes in the environment and to stress.^7^–^8^ Additionally, gonadal sex steroid hormones exert organizational (permanent) and activational (transient) effects on the brain to regulate sexual differentiation, secondary sex characteristics, and sex differences in behavior.^4^,^9^–^11^ Gonadal steroids also influence the stress response that is mediated by the hypothalamic-pituitary-adrenal (HPA) axis, and elevated stress hormones affect the reproductive or hypothalamic-pituitary-gonadal (HPG) axis.^8^ Finally, sex and stress hormones influence alcohol consumption and behavior in models of addiction.^4^–^5^,^10^,^12^ As a result, it should be considered that alcohol consumption can influence the endocrine system and the reciprocal interaction between the stress and reproductive axes and that gonadal and stress steroid hormones can influence alcohol drinking and addiction-related behaviors.

This review highlights preclinical research on the contribution of gonadal and stress steroids to alcohol drinking in females. It focuses on the HPG and HPA axes and describes how endogenous fluctuations in steroid hormones as well as exogenous administration influence alcohol drinking and other pertinent addiction-related phenotypes. In addition to a discussion of how classical steroid responses are mediated by genomic effects via intracellular receptors, this review considers rapid steroid responses via membrane receptors and the interaction with neurotransmitter systems. Relevant comparisons to results in males bolster the emerging evidence for sex differences in steroid hormone and stress effects on alcohol drinking behavior and addiction-related phenotypes. These comparisons emphasize the importance of considering sex in the development of novel pharmacotherapies for the treatment of AUD.

## OVERVIEW OF THE HPG AND HPA AXES

**Figure 1:**
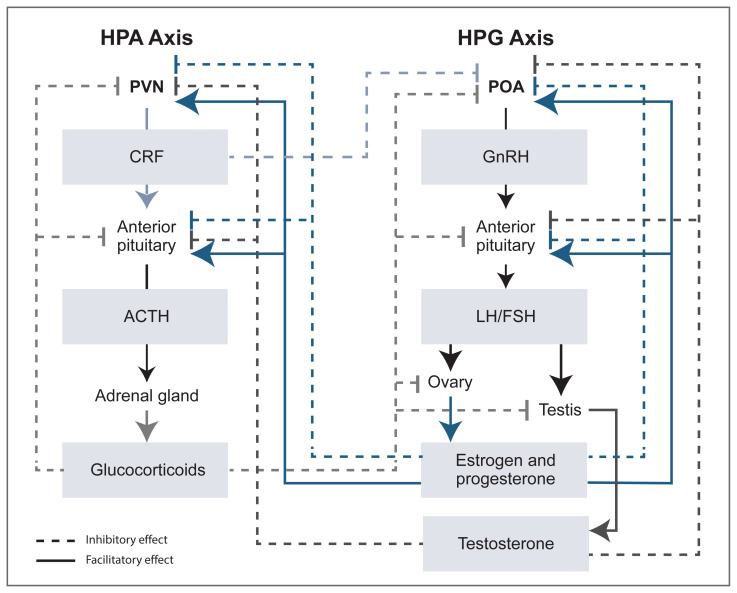
Simplified diagram of the reciprocal interaction between the HPA axis and the HPG axis Solid lines with arrows depict facilitatory effects. Dashed lines with block symbols depict inhibitory or negative feedback effects. Gonadal steroids are involved in the regulation of the HPA axis at the level of the PVN and the anterior pituitary. Specifically, testosterone has negative feedback effects at the PVN and the anterior pituitary, and estrogen and progesterone can have either a facilitatory or an inhibitory effect at the PVN and the anterior pituitary. Stress steroids can regulate the HPG axis at the level of the hypothalamic POA, anterior pituitary, and gonads (ovaries or testes). Glucocorticoids (corticosterone in rodents, cortisol in humans and monkeys) exert negative feedback at each level of the HPG axis, and CRF exerts negative feedback at the POA. Upstream regulatory centers for each axis are not shown. Also shown is the negative feedback exhibited by glucocorticoids within the HPA axis, the negative feedback exhibited by testosterone within the HPG axis, and the negative and positive feedback exhibited by estrogen and progesterone within the HPG axis. *Note:* ACTH, adrenocorticotropic hormone; CRF, corticotropin releasing factor; FSH, follicle stimulating hormone; GnRH, gonadotropin releasing hormone; HPA, hypothalamic-pituitary-adrenal; HPG, hypothalamic-pituitary-gonadal; LH, luteinizing hormone; POA, preoptic area; PVN, paraventricular nucleus. *Source:* Modified from a figure by Oyola and Handa.^8^

The HPG axis is the neuroendocrine axis important for reproduction, whereas the HPA axis is the neuroendocrine axis important for the stress response. As depicted in Figure 1, both the HPG and HPA axes are regulated by steroid hormone feedback and reciprocal interactions between steroids in each axis.

The HPG axis comprises the hypothalamus, pituitary, and gonads. Hypothalamic nuclei (e.g., in the preoptic area) release gonadotropin-releasing hormone (GnRH) into the portal vasculature to stimulate the release of luteinizing hormone (LH) and follicle-stimulating hormone (FSH) from the anterior pituitary (see Figure 1). Circulating LH and FSH act on the gonads to stimulate the production and release of estrogen and progesterone from the ovary and of testosterone from the testis.^7^,^13^ In females, FSH stimulates follicle development in the ovary and the secretion of estradiol, which promotes a surge in LH and FSH. LH stimulates ovulation and the subsequent secretion of progesterone. These overall effects of estradiol are similar across species, but phases of the 28- to 30-day menstrual cycle in primates and the 4- to 5-day estrous cycle in rodents are not completely analogous (see the box **Phases of Primate Menstrual and Rodent Estrous Cycles**). Additionally, steroid hormone feedback loops regulate HPG axis function at the level of the hypothalamus and anterior pituitary. Testosterone inhibits GnRH, LH, and FSH through negative feedback, whereas estradiol and progesterone can exert both negative (inhibitory) and positive (stimulatory) feedback actions, depending on the stage of the ovarian cycle (see Figure 1).

Phases of Primate Menstrual and Rodent Estrous Cycles*

**Table:** 

Primate (Human and Monkey)	Rodent (Rat and Mouse)
The average length of the menstrual cycle is 28 to 30 days.	The average length of the estrous cycle is 4 to 5 days.
**Follicular phase:** As the ovarian follicle develops, estradiol is secreted. Menstruation overlaps with the beginning of the follicular phase.	**Metestrus/diestrus phase:** As the ovarian follicle develops, estradiol is secreted.
**Periovulatory phase:** A rapid estradiol increase triggers an LH surge, which produces ovulation.	**Proestrus/estrus phase:** A rapid estradiol increase triggers an LH surge, which stimulates progesterone release and produces ovulation.
**Luteal phase:** The corpus luteum releases high levels of estradiol and progesterone. Menstruation occurs at the end of the luteal phase as hormone levels fall.	**No equivalent phase:** Female rodents do not have a functional corpus luteum.

* Adapted from a table by Becker and Koob.^4^

*Note:* LH, luteinizing hormone.

Responses to stress are mediated by the HPA axis and the sympathetic autonomic response. Short-term activation of the HPA axis produces beneficial effects, whereas chronic activation can result in deleterious effects.^14^ Neurons in the paraventricular nucleus (PVN) of the hypothalamus are responsible for the secretion of corticotropin releasing factor (CRF) and arginine vasopressin into the portal system, and CRF causes the release of adrenocorticotropic hormone (ACTH) from the anterior pituitary. ACTH stimulates the biosynthesis and release of glucocorticoids from the adrenal cortex.^13^ Negative feedback of glucocorticoids at the level of the anterior pituitary and PVN inhibits CRF, arginine vasopressin, and ACTH production and helps maintain optimal glucocorticoid levels (Figure 1).

An additional consideration is that the HPA and HPG axes have reciprocal interactions in terms of steroid hormone feedback, as depicted in Figure 1.^8^ For example, glucocorticoids exhibit negative feedback of the HPG axis at the level of the hypothalamus, anterior pituitary, and gonads. As a result, a chronic elevation of glucocorticoids can result in suppressed HPG axis function. Likewise, gonadal steroids may influence HPA axis function, as evidenced by the effects of testosterone, progesterone, and estrogen at the level of the PVN and anterior pituitary.^13^ For example, basal and stress-induced increases in glucocorticoids are greater in female than in male rodents. Evidence from studies that used gonadectomy and hormone replacement suggests that testosterone exerts an inhibitory influence on HPA axis activity in male rodents, whereas estrogen primarily produces a facilitatory effect on HPA axis activity in female rodents. Some of the differing results for estrogen on HPA axis function may be due in part to the opposing actions of two types of estrogen receptors.^13^

## STEROID HORMONE RECEPTORS AND CIRCUITRY IMPORTANT FOR STRESS AND DRINKING

Steroid hormones produce effects through several mechanisms. First, steroid hormones bind to their classical intracellular receptors, which act as ligand-activated transcription factors to alter gene expression and produce long-lasting actions.^13^ Progestins, such as progesterone and dihydroprogesterone, bind to two progesterone receptor isoforms: A and B.^15^ Estrogens, such as 17beta-estradiol, bind to two distinct receptor subtypes: estrogen receptor-alpha and estrogen receptor-beta.^13^,^16^ Androgens, such as testosterone and dihydrotestosterone, bind to androgen receptors.^13^ Glucocorticoids, such as corticosterone in rodents and cortisol in humans and monkeys, bind to mineralocorticoid receptors (type I) and glucocorticoid receptors (type II).^13^ Endogenous glucocorticoids have higher affinity for mineralocorticoid receptors than for glucocorticoid receptors.^13^

Second, through classical and nonclassical receptors located in the cell membrane, steroids have rapid effects that influence second-messenger pathways and ion channel function.^16^–^22^ Finally, steroid hormone derivatives can rapidly alter ion channel function via allosteric interactions with ligand-gated ion channels.^23^–^26^ For example, the progesterone derivative allopregnanolone and the deoxycorticosterone derivative tetrahydrodeoxycorticosterone (THDOC) are very potent positive allosteric modulators of gamma-aminobutyric acid Subscript A (GABA Subscript A ) receptors and can rapidly alter neuronal inhibition. Rapid actions at the cell membrane gave rise to the terms “neuroactive steroids” and “neurosteroids” (Refer to the Finn and Jimenez article on neurosteroid networks for more information about neurosteroid synthesis and pathways.)^24^ Thus, steroid hormones and their derivatives can influence brain function and behavior through classic genomic actions and rapid membrane effects.

**Figure 2:**
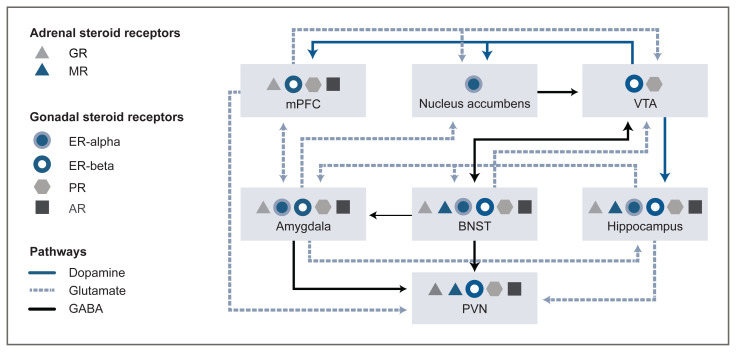
Simplified stress and mesocorticolimbic circuitry, including inputs to the HPA axis and the distribution of gonadal and adrenal steroid receptors Rapid steroid actions at associated receptors and neurosteroid actions at GABA Subscript A receptors represent additional mechanisms for fine-tuning central nervous system excitability. Gonadal and adrenal steroid receptors have considerable overlap in expression within the hypothalamic (PVN) and extrahypothalamic (e.g., amygdala, BNST) stress circuitry, as well as among components of the mesocorticolimbic (e.g., mPFC, nucleus accumbens, VTA, and hippocampus) circuitry, which ultimately can affect output of the PVN (i.e., the stress response) and alcohol drinking. This simplified circuitry shows GABAergic (red), glutamatergic (green), and dopaminergic (blue) projections within the brain regions that input to the PVN, either directly or indirectly through an inhibitory projection from the peri-PVN (which contains ER-alpha and GR, not shown). The brain regions involved and the overall influence on the output of the PVN (and HPA axis activity) depend on the stressor modality, the level of acute or chronic alcohol consumption, and the various steroid and neurosteroid levels and actions at their associated receptors. *Note:* AR, androgen receptor; BNST, bed nucleus of the stria terminalis; ER-alpha, estrogen receptor-alpha; ER-beta, estrogen receptor-beta; GABA, gamma-aminobutyric acid; GR, glucocorticoid receptor; HPA, hypothalamic-pituitary-adrenal; mPFC, medial prefrontal cortex; MR, mineralocorticoid receptor; PR, progesterone receptor (both isoforms); PVN, paraventricular nucleus; VTA, ventral tegmental area. *Source:* Circuitr*y*^13^,^24^ and steroid receptor distribution^13^,^15^,^21^,^33^–^36^ are modified from other sources.

Neuroanatomical overlap occurs between gonadal and adrenal steroid hormone receptors within the hypothalamic (the PVN) and extra-hypothalamic (e.g., in the amygdala and the bed nucleus of the stria terminalis) stress circuitry (see Figure 2). Overlap also occurs within components of the mesocorticolimbic circuitry (e.g., in the medial prefrontal cortex, nucleus accumbens, ventral tegmental area, and hippocampus). Ultimately, this overlap can affect output of the PVN (i.e., the stress response) and alcohol drinking. Figure 2 shows simplified circuitry of glutamatergic, GABAergic, and dopaminergic projections in brain regions important for responses to stress and alcohol drinking behavior. These responses to stress and alcohol drinking behavior may be modulated by steroid actions at receptors localized within the brain regions.

For example, the brain regions involved and the overall influence on PVN output depends on the stress, on various steroid hormone levels and actions at associated receptors,^8^,^13^ and on GABA Subscript A receptor–active neurosteroid levels and actions at GABA Subscript A receptors.^24^ Alcohol’s ability to activate the HPA axis relies on activation of the PVN.^27^ Synaptic connections within the PVN are primarily GABAergic and glutamatergic.^28^,^29^ As a result, glutamatergic afferents in the forebrain that increase GABA release in the PVN, and upstream GABAergic projection neurons that activate the PVN, produce tonic inhibition of the PVN.^30^

Additionally, stress-induced elevations in GABA Subscript A receptor–active neurosteroids can modulate PVN activity, given that physiological concentrations of allopregnanolone (i.e., 10 nM to 100 nM) inhibit output of PVN neurons (i.e., CRF release) via a potentiation of GABA Subscript A receptors. 31,32 A neurosteroid-induced inhibition of CRF release likely represents another mechanism for terminating the stress response.

Another consideration is that alcohol-induced alterations to neurotransmission within the circuitry depicted in Figure 2 can be modulated by steroid hormone and neurosteroid levels. For instance, estradiol and progesterone can rapidly affect dopamine signaling via actions at their respective steroid receptors, functional coupling between estrogen receptors (both alpha and beta) and metabotropic glutamate receptors (Group I or Group II) can activate distinct signaling pathways, and neurosteroids can rapidly increase GABA Subscript A receptor–mediated signaling.^21^,^23^,^24^,^33^–^36^ Thus, rapid steroid actions at associated receptors and neurosteroid actions at GABA Subscript A receptors are other mechanisms for fine-tuning central nervous system excitability.

## STEROID HORMONE EFFECTS ON DRINKING AND OTHER ADDICTION-RELATED BEHAVIORS

Investigations of sex differences in drug misuse and self-administration behavior have gained momentum, particularly after 2015, when the National Institutes of Health announced a policy of including sex as a biological variable. Clinical and preclinical alcohol research offers many examples of sex differences, given that alcohol exposure can produce sexually dimorphic effects throughout life. Discussion of all these studies is beyond the focus of this review, but several excellent reviews describe sex differences in the effects of alcohol exposure across development. Reviews have summarized findings from prenatal^37^ and adolescent^38^–^41^ alcohol exposure, as well as from exposure during adulthood.^4^,^7^ Marked sex differences in self-administration patterns have been well-documented and observed at every stage of the course of drug exposure, from acquisition to maintenance to relapse, although more evidence has been reported for psychostimulants than for alcohol.^42^,^43^

In general, results from preclinical alcohol models indicate that females acquire self-administration of alcohol more rapidly and consume larger alcohol doses during maintenance phases than males, but females exhibit a reduced severity in somatic and negative affective symptoms of alcohol withdrawal than males.^4^ Although the potential role of organizational steroid effects in controlling sex differences in alcohol responses cannot be ruled out, this review focuses primarily on the effects, during adulthood, of estrogen, progesterone, and neuroactive metabolites on alcohol drinking and pertinent addiction-related phenotypes in females.

### Gonadal Steroids

In a variety of models of alcohol access, preclinical research in rodents documents that females consume larger doses of alcohol than males. This sex difference appears to be partly due to a facilitatory effect of estrogen in females and an inhibitory effect of testosterone in males.^4^,^44^ In female rodents, the estrous cycle phase had minimal effects on alcohol drinking or operant self-administration.^45^ Reduced self-administration of alcohol was observed in females during proestrus and estrus only when their cycles had been experimentally synchronized (the effect was not observed in randomly cycling females that were not synchronized). Likewise, microanalysis of alcohol drinking patterns revealed increased frequency of bouts but less alcohol consumed within each bout during proestrus,^46^ suggesting subtle differences in the pattern of alcohol drinking across the estrous cycle. In several models, more recent evidence confirmed that the phase of estrous cycle did not significantly influence alcohol drinking, including binge drinking,^47^ escalated drinking among dependent animals,^48^ self-administration of alcohol,^49^ or cue plus yohimbine-induced reinstatement of alcohol-seeking.^49^

In contrast to studies of rodents, a recent, longitudinal study of female rhesus monkeys with systematic and extensive hormonal monitoring of menstrual cycle phase across 15 months of active alcohol drinking determined that the monkeys drank more alcohol during the luteal versus the follicular phase and drank the most alcohol during the late luteal phase, when progesterone declines rapidly.^50^ These results from a nonhuman, primate model of self-administration of alcohol were the first to show that typical menstrual cycle–related fluctuations in progesterone, especially during the late luteal phase, modulated alcohol drinking. Previous studies that used less accurate characterization of menstrual cycles and differing histories of alcohol intake revealed inconsistent effects of the menstrual cycle on alcohol drinking. Therefore, Dozier and colleagues’ method of extensive menstrual cycle characterization during periods of active drinking^50^ likely was necessary to show the significant menstrual cycle–related fluctuation in alcohol drinking.

The results by Dozier and colleagues are consistent with clinical studies in which increases in premenstrual distress and negative affective states in women were positively correlated with greater alcohol drinking during the late luteal phase.^4^,^51^ Thus, existing data support the conclusion that typical hormonal fluctuations during the menstrual cycle, but not during the estrous cycle, can influence alcohol drinking. These differences may reflect hormonal changes during the menstrual cycle that are distinct from those in the estrous cycle,^51^ because rodents have no equivalent luteal phase (see the box **Phases of Primate Menstrual and Rodent Estrous Cycles**).

Despite minimal effects of the estrous cycle phase on alcohol drinking, several lines of evidence in studies of rodents indicate that the hormonal milieu contributes to sex differences in models of alcohol drinking behavior and alcohol reward. First, development of the four core genotype (FCG) mouse model has enabled researchers to examine the sex chromosome complement (XX versus XY) and the gonadal phenotype (testes versus ovaries) and their independent contributions to sex differences.^52^ This model produces four different progeny, each with a different combination of sex chromosomes and gonadal sex: XXF (XX gonadal females), XXM (XX gonadal males), XYF (XY gonadal females), and XYM (XY gonadal males). Use of the FCG model determined that gonadal phenotype predicted self-administration of alcohol, independent of the sex chromosome complement.^53^ That is, gonadal females consumed more alcohol than gonadal males.

Second, several studies that used gonadectomy and hormone replacement found that when compared with intact female rats, female rats with gonadectomy drank significantly less alcohol.^54^,^55^ After the gonadectomized rats received estradiol replacement, the low levels of alcohol drinking increased significantly to baseline levels. Also, in female mice, gonadectomy significantly reduced binge drinking from the high levels of consumption among intact females to levels of consumption equivalent to that of intact males.^47^ The lower levels of binge drinking among female mice with gonadectomy increased significantly following replacement with 17beta-estradiol.^47^

Similarly, gonadectomy in male and female rats produced shifts in operant alcohol self-administration toward the pattern of the opposite sex (i.e., reduced for females and increased for males).^49^ In these rats, estradiol replacement in females with gonadectomy significantly increased self-administration of alcohol, and testosterone replacement in males with gonadectomy significantly decreased self-administration of alcohol. However, in rodent males, the suppressive effect of testosterone on alcohol drinking contrasts with fairly consistent clinical reports that found positive associations between blood or salivary testosterone levels and alcohol drinking among human adolescent and adult males.^10^

Third, in studies that used conditioned place preference as a measure of alcohol reward, only intact female rats exhibited conditioned place preference to an intermediate alcohol dose.^56^ Intact male rats and female rats with gonadectomy (males with gonadectomy were not tested) did not exhibit the preference for the drug paired side of the testing chamber. Subsequent studies in female mice determined that in females with gonadectomy, 17beta-estradiol facilitated alcohol-induced conditioned place preference due to activation of both estrogen receptor-alpha and estrogen receptor-beta.^57^

The facilitatory effects of estradiol on alcohol drinking and a measure of alcohol reward may be due, in part, to estradiol’s rapid enhancement of dopaminergic signaling.^36^ In the prefrontal cortex, the ability of a low dose of alcohol (0.5 g/kg) to enhance extracellular dopamine levels in female rats during estrus was eliminated by gonadectomy and restored by estradiol treatment.^58^ In the striatum, the well-documented ability of estradiol to enhance dopaminergic signaling in females was hypothesized to be associated with effects of estradiol on membrane-localized estrogen receptor-alpha and estrogen receptor-beta that were functionally coupled to metabotropic glutamate receptors.^34^,^36^ Collectively, research confirms that within each sex, activational effects of gonadal steroids can modulate alcohol drinking behavior.

The organizational effect of testosterone-derived estrogen, which causes sex-specific differentiation of the mammalian brain,^9^,^52^,^59^ during a critical period of brain development, also influences alcohol drinking. Early work found that neonatal exposure to estrogen among female rats, which conferred a male phenotype on a genetically female brain, produced levels of alcohol drinking that were lower than levels in intact females but similar to levels in intact males.^60^

More recent work has determined that gonadectomy alone in male and female rats shifted self-administration of alcohol toward the pattern of the opposite sex, but it did not eliminate the sex difference.^49^ Females with gonadectomy still self-administered more alcohol than males with gonadectomy. Likewise, during tests of alcohol-seeking (cue plus yohimbine-induced reinstatement), intact females engaged in active lever presses more than intact males. Females with gonadectomy still had more lever presses than males with gonadectomy, and lever presses were not altered by steroid replacement (i.e., estradiol in females and testosterone in males). These results suggest that in addition to the contribution of the activational effects of gonadal steroids on alcohol drinking in males and females, permanent factors, such as sex chromosomes and the organizational effects of gonadal steroids, contribute to sex differences in alcohol-drinking and alcohol-seeking behaviors.

Use of the FCG model also determined that independent of gonadal phenotype, the sex chromosome complement mediates habitual responding for alcohol reinforcement after moderate instrumental training.^53^ Specifically, XY mice (XYM and XYF) were insensitive to alcohol devaluation, a procedure that established conditioned taste aversion by pairing alcohol consumption with lithium chloride injections. Both valued (no conditioned taste aversion) and devalued (with conditioned taste aversion) XY mice responded similarly, indicating that XY mice were responding in a habitual manner. XX mice (XXM and XXF) were sensitive to alcohol devaluation (devalued XX mice responded less than valued XX mice), indicating that XX mice retained goal-directed responding.^53^

Given that AUD involves a transition from casual to habitual use, as well as a transition from ventral striatal circuitry including the prefrontal cortex to a more dorsal circuit involving the dorsolateral striatum,^61^ the results from Barker and colleagues^53^ suggest that sex chromosomes mediate sex differences in habit formation for alcohol, and they may underlie sex differences in alcohol-induced neuroadaptation. Additional studies are necessary to disentangle the contribution of sex chromosomes and the organizational effects of gonadal steroids on alcohol-motivated behavior.

### Neurosteroids

Studies have examined whether manipulation in levels of the progesterone derivative allopregnanolone, which is a potent, positive allosteric modulator of GABA Subscript A receptors,^23^–^26^ alters alcohol drinking and alcohol’s subjective effects. In general, females have higher endogenous allopregnanolone levels than males. Allopregnanolone levels in females fluctuate across the estrous and menstrual cycles and increase during pregnancy in a time-dependent manner that is related to fluctuations in endogenous progesterone.^25^,^62^,^63^ The majority of studies, which were conducted in male rodents, consistently have shown that allopregnanolone, after systemic and intracerebroventricular administration, exerts a biphasic effect (i.e., increases with low physiological doses and decreases with supraphysiological doses) on alcohol drinking and operant self-administration.^64^

**Figure 3:**
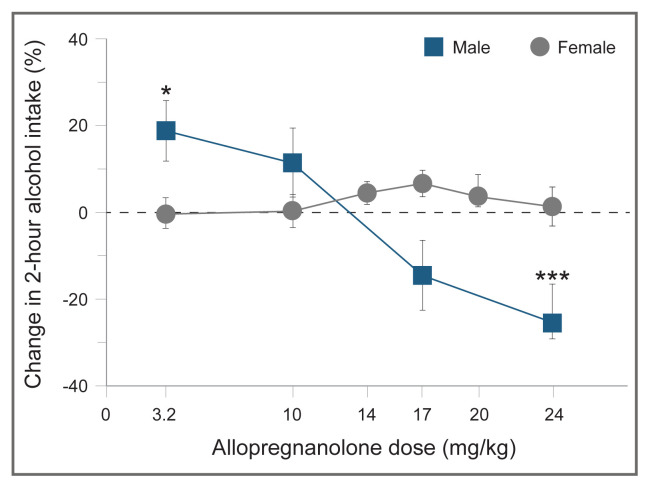
Sex differences in the modulatory effect of allopregnanolone on limited-access alcohol drinking in mice Dose response is shown as a percentage of change from baseline values (vehicle treatments). The graph depicts the means and standard errors for 18 male and 24 female C57BL/6J mice. The dashed line represents the baseline values. *Note:* *p ≤ 0.05; ***p ≤ 0.001 versus respective vehicle treatment (20% beta-cyclodextrin). *Source:* Adapted from Finn DA, Beckley EH, Kaufman KR, et al.^64^

In contrast, research has shown that allopregnanolone does not alter alcohol drinking in female mice (see Figure 3).^65^ Administration of the 5alpha-reductase inhibitor finasteride to mice, which decreased endogenous GABA Subscript A receptor–active neurosteroids such as allopregnanolone,^65^ produced a decrease in the acquisition and maintenance phases of self-administration of alcohol in males, with females, again, being less sensitive to these modulatory effects.^66^–^68^ A priming dose of allopregnanolone promoted reinstatement of alcohol-seeking behavior in male mice and rats,^69^,^70^ but similar studies in females have not been conducted.

Finally, evidence also suggests that allopregnanolone and its 5beta-isomer, pregnanolone, like alcohol, possess positive motivational effects, as demonstrated by conditioned place preference among male mice,^71^ preference for drinking steroids versus water in male mice and rats,^72^,^73^ and intravenous self-administration in four rhesus monkeys, with the highest self-administration of pregnanolone in the one female versus the three male monkeys.^74^ Both allopregnanolone and pregnanolone produced potent, alcohol-like, discriminative stimulus effects in male and female cynomolgus monkeys.^75^ Also, during the luteal phase of the menstrual cycle, when endogenous allopregnanolone levels were highest, female cynomolgus monkeys were more sensitive to the discriminative stimulus effects of alcohol and to the alcohol-like effects of allopregnanolone.^76^ Collectively, these results suggest that GABAergic neurosteroid levels may enhance the reinforcing effects of alcohol, and that in rodents, sensitivity to neurosteroid effects differs by sex.

A comparison of results in female mice and monkeys suggests that female monkeys are more sensitive to allopregnanolone’s modulatory effects on alcohol drinking behavior. However, the relative insensitivity in female mice contrasts with the enhanced sensitivity to the anticonvulsant effect of allopregnanolone and THDOC during alcohol withdrawal in female rats and in female mice that have a low withdrawal phenotype.^77^–^79^

**Figure 4:**
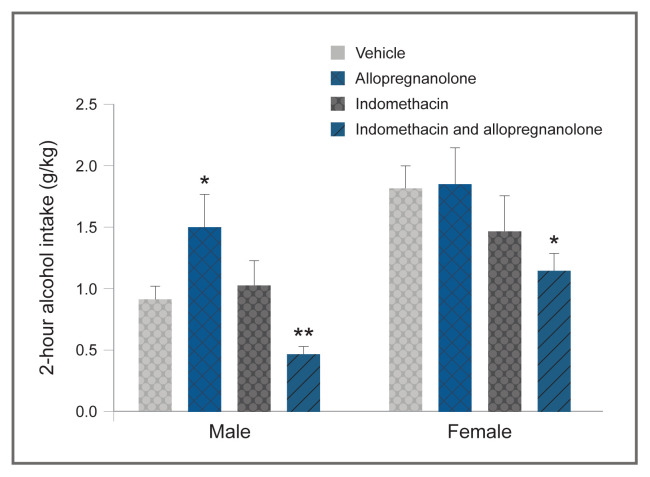
Modulatory effect of a combination of allopregnanolone and indomethacin in male and female mice Female mouse insensitivity to allopregnanolone’s modulatory effect on limited-access alcohol drinking was overcome by administering 0.1 mg/kg indomethacin along with 10 mg/kg allopregnanolone. Indomethacin blocks the oxidation of allopregnanolone and thereby enhances allopregnanolone’s effect on GABA Subscript A receptor–mediated inhibition. The graph depicts the means and standard errors for 10 male and 10 to 11 female C57BL/6J mice. *Note*: *p ≤ 0.05; **p ≤ 0.01 versus respective vehicle treatment (20% beta-cyclodextrin). *Source:* DA Finn and MM Ford, unpublished data, May 2013.

Based on evidence that local allopregnanolone metabolism in hippocampal subregions significantly altered GABA Subscript A receptor–mediated inhibition,^80^ a sex difference in allopregnanolone metabolism in discrete brain regions in mice possibly contributes to low sensitivity to allopregnanolone’s modulatory effects on alcohol drinking. Belelli and Herd used the 3alpha-hydroxysteroid dehydrogenase (3alpha-HSD) inhibitor indomethacin to inhibit oxidation of allopregnanolone to dihydroprogesterone, which increased local allopregnanolone levels and enhanced GABA Subscript A receptor–mediated inhibition.^80^ Early work indicated that female rats, when compared with males, had about twice the activity of 3alpha-HSD from rat-liver cytosol, and that this sex difference was induced by ovarian estrogen.^81^ So, in female rodents, more 3alpha-HSD activity within neurocircuitry fundamental to the regulatory processes underlying alcohol intake possibly contributes to insensitivity to the effects of allopregnanolone on alcohol drinking. Consistent with this idea, administration of allopregnanolone and indomethacin in female mice did not alter alcohol drinking when administered separately but produced a significant decrease in alcohol drinking when administered in combination (see Figure 4, DA Finn and MM Ford, unpublished data, May 2013).

**Figure 5:**
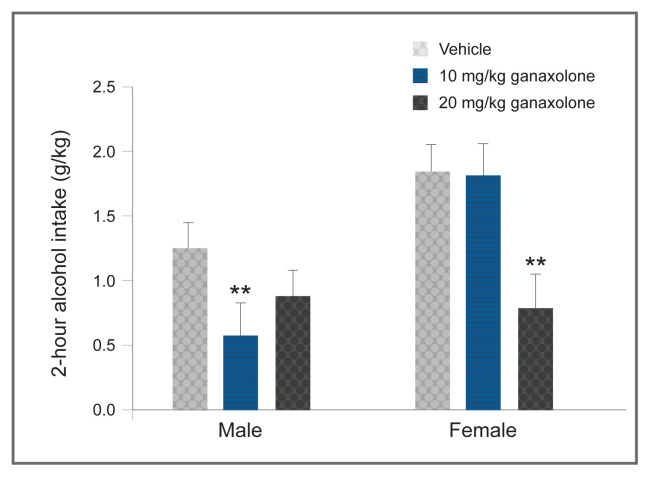
Sex differences in the modulatory effect of the synthetic neurosteroid ganaxolone in mice Ganaxolone significantly decreased limited-access alcohol drinking in males and females. To significantly suppress alcohol drinking, female mice required a higher dose (20 mg/kg) than male mice (10 mg/kg). The graph depicts the means and standard errors for 10 male and 10 to 11 female C57BL/6J mice. *Note:* **p ≤ 0.01 versus respective vehicle treatment (20% beta-cyclodextrin). *Source:* DA Finn and MM Ford, unpublished data, April 2013.

Another strategy for avoiding potential confounds of rapid allopregnanolone metabolism is use of a synthetic allopregnanolone analog, such as ganaxolone.^82^ Ganaxolone has a similar pharmacological profile to allopregnanolone, but it has an additional 3beta-methyl group that protects the steroid from metabolic attack at the 3alpha-position and extends the half-life about three to four times longer than that of allopregnanolone. In male rodents, ganaxolone produced a biphasic effect on alcohol drinking and self-administration when administered systemically^83^–^85^ or bilaterally into the nucleus accumbens shell.^86^ Systemic ganaxolone also promoted reinstatement of alcohol-seeking,^87^ These effects of ganaxolone on alcohol drinking and seeking were similar to those observed following allopregnanolone administration. Preliminary results suggest that ganaxolone also significantly reduces alcohol drinking in female mice, although a higher dose was required to produce a comparable reduction to that observed in male mice (see Figure 5, DA Finn and MM Ford, unpublished data, April 2013).

The U.S. Food and Drug Administration recently approved the allopregnanolone analog brexanolone for treatment of postpartum depression. In addition, ganaxolone is in phase 2 clinical trials for treatment of various disorders, such as postpartum depression, treatment-resistant depression, post-traumatic stress disorder (PTSD), and epilepsy. Allopregnanolone analogs and strategies to stabilize allopregnanolone levels also are being examined in clinical trials for the treatment of various central nervous system disorders.^88^ Collectively, evidence suggests that targeting neurosteroid synthesis or use of neurosteroid analogs such as ganaxolone may represent innovative therapies for the treatment of AUD in males and females.^26^

## EFFECTS OF CHRONIC ALCOHOL USE ON GONADAL STEROID LEVELS

Alcohol misuse and AUD produce significant hormonal disruptions in the endocrine system.^7^ For sex steroids, the majority of evidence in rodents and humans suggests that chronic alcohol exposure significantly increases estradiol levels in both males and females, produces a slight or significant decrease in progesterone levels in both males and females, decreases testosterone levels in males, and produces a transient increase in testosterone levels in females. Additional work found that chronic exposure to alcohol vapor to induce dependence significantly increased testosterone levels in female mice and suggested that the increased testosterone levels in dependent female mice contributed to an observed estrous cycle disruption (i.e., prolonged diestrus).^89^

Thus, the HPG dysfunction that occurs in people with AUD can be associated with deleterious effects on reproduction in both males and females. However, some preclinical studies suggest that 6 weeks of binge drinking by female rodents^47^ or 15 months of active drinking by female monkeys^50^ did not significantly alter the estrous or menstrual cycles, respectively, in terms of overall cycle length or the length of specific cycle phases. Fifteen months of active drinking also did not alter progesterone or estradiol levels in the female monkeys.^50^ The method of chronic alcohol exposure and resulting blood alcohol concentrations, which are considerably higher for vapor exposure (e.g., 200 mg%) than for drinking models (e.g., 80 mg% to 100 mg%), may contribute to the differences among studies with regard to whether chronic alcohol exposure disrupted the estrous or menstrual cycle.

## EFFECTS OF CHRONIC ALCOHOL USE ON NEUROSTEROID LEVELS

Preclinical models of chronic alcohol drinking and vapor exposure both produce significant alterations in neurosteroid levels. Most of the evidence supports changes to allopregnanolone levels in plasma and in discrete brain regions.^24^ The majority of available data are from studies in male rodents and monkeys. The results consistently show that chronic alcohol drinking and vapor exposure significantly decrease plasma allopregnanolone levels during acute withdrawal, a finding in harmony with the limited results reported for males and females with AUD.

In a small cohort of females with AUD, a significant reduction in allopregnanolone, progesterone, and estradiol levels was detected upon detoxification, and levels recovered to baseline values after 4 months of abstinence.^90^ In contrast, chronic alcohol drinking did not significantly alter serum allopregnanolone levels in female monkeys,^50^ nor did withdrawal from chronic alcohol vapor exposure alter plasma allopregnanolone levels in female mice (DA Finn and JP Jensen, unpublished data, Feb 2019 and Nov 2019).

Regarding brain regional changes, chronic alcohol exposure and withdrawal significantly decreased allopregnanolone levels in the amygdala of male monkeys and in the nucleus accumbens, ventral tegmental area, and medial prefrontal cortex of male rodents, with divergent changes reported in hippocampal subregions in male rodents.^24^ However, preliminary results in female mice suggest that withdrawal from chronic alcohol exposure did not significantly alter cortical or hippocampal allopregnanolone levels (DA Finn and JP Jensen, unpublished data, Feb 2020 and Mar 2020).

Collectively, preclinical results in male rodents and monkeys suggest that independent adrenal and brain region regulation of neurosteroid synthesis occurs after chronic alcohol exposure and withdrawal. More preclinical research in females is necessary, but the available preclinical results suggest that females may be protected from chronic alcohol–induced suppression of allopregnanolone synthesis. Given the preclinical evidence that severity of alcohol withdrawal is reduced in females versus males,^4^ and that allopregnanolone has anticonvulsant, anxiolytic, and antidepressant properties,^24^ females may have the ability to maintain endogenous allopregnanolone levels after chronic alcohol exposure. This maintenance, versus the suppression seen in males, may contribute to the female phenotype for reduced severity and duration of alcohol withdrawal.

## STRESS STEROIDS AND ALCOHOL-RELATED BEHAVIOR

Clinical studies provide evidence for a positive association between stress and alcohol drinking and other phases of AUD, including evidence of stress as a trigger of alcohol relapse.^91^ Additionally, males and females have different sensitivities to alcohol and stress.^4^–^6^ Acute stress exposure and alcohol intoxication both activate the HPA axis, and the HPA and HPG interact reciprocally (Figure 1).^8^ Therefore, sex differences in HPA axis responsivity following acute stress or acute alcohol intoxication (i.e., enhanced elevation in glucocorticoids in females versus males) are not surprising. Discussion of all studies on this topic is beyond the scope of this review, but other reviews provide more detail.^5^,^8^,^13^,^92^

Preclinical studies demonstrate conflicting evidence regarding the influence of various stressors on alcohol drinking in rodents, and sex- and stress-related alterations in drinking vary with the stress model used.^5^,^93^ However, a few examples of results show a sex difference in the relationship between corticosterone levels and alcohol drinking or alcohol-seeking.

First, studies have shown that exposure to predator odor stress (PS), which is considered a traumatic stress and used as a model of PTSD, significantly increases alcohol drinking and self-administration in rodents.^94^ Evidence supports greater PS-enhanced drinking among female versus male mice.^93^,^95^ Plasma corticosterone levels following PS exposure have been shown to be significantly higher in female versus male mice when mice were naïve and also when the mice had a history of alcohol drinking.^93^,^95^ Also, investigators have reported a significant positive correlation between plasma corticosterone levels and alcohol intake on the first day after PS exposure. When all mice were considered, the goodness of fit of the regression line (*R* Superscript 2 = 0.26, *p* < 0.05) indicated that the variation in PS-induced corticosterone levels accounted for 26% of the variance in alcohol drinking on the day after PS exposure. The relationship was stronger in females (*R* Superscript 2 = 0.42, *p* < 0.05), confirming that the amount of HPA axis activation after PS exposure significantly influenced alcohol drinking the following day.^93^

Second, studies examining cue plus yohimbine-induced reinstatement of alcohol-seeking in male and female rats determined that active lever presses during the reinstatement tests were significantly higher in females versus males.^96^ During the reinstatement testing for female rats only, corticosterone and estradiol levels were significantly, positively correlated with active lever presses.^96^

Third, in mice deficient in beta-endorphin (knockout mice), a peptide that regulates HPA axis activity via mu opioid receptor–mediated inhibition, the females had elevated basal levels of anxiety, plasma corticosterone, and CRF in the extended amygdala when they were compared with female wild-type mice.^97^ High binge alcohol intake in the female beta-endorphin knockout mice normalized their high levels of basal anxiety, corticosterone, and CRF. This relationship was not observed for the male beta-endorphin knockout mice when they were compared with wild-type mice.

Fourth, in mice with a history of alcohol drinking and exposure to PS, the PS-induced increase in plasma corticosterone was significantly lower in male mice, and tended to be lower in female mice, versus respective naïve mice.^95^ This result is consistent with evidence that AUD in humans and alcohol dependence in rodents can lead to a dampened neuroendocrine state in terms of HPA axis responsivity.^7^ Collectively, the results suggest that overlapping stress and gonadal steroids, as well as sex differences in HPA axis responsivity, contribute to sex differences in alcohol drinking, alcohol-seeking, and interaction with stress.

Preclinical studies also demonstrate cellular and molecular sex differences in stress response systems.^5^,^8^,^13^,^92^ Both glucocorticoid receptors and CRF Subscript 1 receptors are being pursued as potential targets for AUD pharmacotherapies, but preclinical data in support of these targets have been generated primarily in males.^98^ Recent work in male and female mice found that a history of alcohol drinking and intermittent PS exposure produced sexually divergent and brain region differences in protein levels for glucocorticoid receptors and CRF Subscript 1 receptors.^95^ Increased cortical glucocorticoid receptor levels and hippocampal CRF Subscript 1 receptor levels were only found in female mice. These findings are consistent with evidence for impaired glucocorticoid negative feedback resulting from inhibition of glucocorticoid receptor translocation and evidence for increased CRF Subscript 1 receptor signaling and decreased CRF Subscript 1 receptor internalization in female versus male rodents.^92^

Collectively, an increased endocrine response to stress and alcohol consumption in females may result from sex differences that occur at the molecular and systems level. The sex differences in CRF Subscript 1 receptor and glucocorticoid receptor protein levels described above suggest that sexually divergent mechanisms may contribute to HPA axis dysregulation following a history of alcohol drinking and repeated stress exposure. As a result, pharmacological strategies targeting the CRF Subscript 1 receptor and glucocorticoid receptor systems may be differentially effective in males versus females.

## EFFECTS OF STRESS ON NEUROSTEROID LEVELS

Exposure to stress^31^ and models of acute alcohol intoxication^24^,^99^ also significantly increase levels of GABA Subscript A receptor–active neurosteroids, although some species differences in the effects of alcohol administration on neurosteroid levels have been reported.^100^ In addition, most of these studies were conducted in males. In male rats, alcohol’s steroidogenic effect was shown to be regulated by an alcohol-induced increase in ACTH release and by de novo synthesis of adrenal steroidogenic acute regulatory protein.^101^ Chronic alcohol exposure blunts alcohol’s steroidogenic effect on neurosteroid levels, but administration of ACTH restores the steroidogenic effect.^102^ Although comparable studies have not been conducted in females, limited data have indicated that CRF and ACTH tests in women significantly increase serum allopregnanolone, progesterone, and dehydroepiandrosterone levels.^63^ Studies also have reported that binge alcohol intoxication in male and female adolescent humans significantly increased serum allopregnanolone levels.^103^,^104^

Preclinical studies found that exposure to various stressors significantly increased plasma allopregnanolone levels in male and female mice that had been consuming alcohol for weeks,^93^ whereas weeks of alcohol consumption alone (i.e., without stress exposure) significantly increased brain allopregnanolone levels in male mice but not in female mice.^62^ Thus, data available for females suggest that stress and activation of the HPA axis increases neurosteroid levels, whereas acute alcohol administration produces inconsistent effects. Additional studies in females are necessary to determine whether an alcohol-induced steroidogenic effect can exert a protective effect against further alcohol drinking, as has been proposed for males.^99^

Two studies with small cohorts of male and female patients with co-occurring AUD and cocaine use disorder found that progesterone administration decreased cue-induced craving and cortisol responses.^105^ The male and female subjects with the highest allopregnanolone levels after progesterone administration showed the greatest reductions in craving,^106^ with no sex differences in these relationships. Consequently, despite no direct data on neurosteroid treatment in patients with AUD, strategies to enhance levels of GABA Subscript A receptor–active neurosteroids, such as allopregnanolone, may represent a biomarker of treatment efficacy among men and women.^5^,^91^

## CONCLUSION

The current review considered the contribution of the endocrine system to alcohol drinking and addiction-related behaviors in females, with a focus on the HPG and HPA axes and their reciprocal interactions. The majority of results from preclinical models indicate that females acquire self-administration of alcohol more rapidly and consume higher alcohol doses during maintenance phases than males. However, aspects of alcohol withdrawal, especially somatic and some negative affective symptoms, are less severe in females than in males. Some of these behavioral differences are due to the organizational and activational effects of gonadal steroids.

Numerous studies that used gonadectomy and steroid replacement documented that gonadal steroids have activational effects and that these activational effects contribute to the higher alcohol drinking, self-administration, and responding during reinstatement tests of alcohol-seeking in females versus males. However, additional studies determined that permanent factors, such as sex chromosomes and the organizational effects of gonadal steroids, also can contribute to sex differences in alcohol drinking and alcohol-seeking. For example, elegant studies that used the FCG mouse model determined that the sex chromosome complement mediated habitual responding for alcohol reinforcement. Additional studies are necessary to distinguish how sex chromosomes and the organizational effects of gonadal steroids contribute to alcohol-motivated behavior.

Sex steroids also influence the stress response, and elevated glucocorticoids can suppress HPG axis function (Figure 1). In addition to the facilitatory and inhibitory feedback mechanisms within and between the HPA and HPG axes, steroid hormones and their derivatives (e.g., neurosteroids) can influence brain function and behavior through classic genomic actions and rapid membrane effects at receptors localized within brain regions important for stress responses and for alcohol-related behaviors (Figure 2). For example, ovarian steroids can modulate dopamine signaling and distinct signaling pathways through actions at their membrane receptors, and neurosteroids can rapidly increase GABASubscript A receptor–mediated signaling. These effects represent another way that steroids and neurosteroids modulate alcohol-drinking and -seeking behaviors.

Likewise, sex steroids modulate PVN output (e.g., the stress response). Estrogen has a facilitatory effect, and testosterone has an inhibitory effect. These effects are consistent with enhanced HPA axis responsivity and elevated glucocorticoids in females versus males. In both sexes, a neurosteroid-induced inhibition of CRF release via enhancement of GABAergic inhibition likely is a mechanism for terminating the stress response.

Another consideration is that the well-documented effects of chronic alcohol use and exposure on steroid levels provides another level of complexity toward understanding the influence of gonadal and stress steroids on alcohol-related behaviors.

Evidence for a positive association between stress and alcohol drinking is strong in clinical studies and mixed in preclinical studies. However, stress is a potent trigger of alcohol relapse in clinical studies and of alcohol-seeking in preclinical studies. HPA axis responsivity is enhanced in females versus males. So, it is interesting that only female rodents exhibited positive correlations between corticosterone levels following stress and stress-enhanced drinking as well as between corticosterone and estradiol levels and lever presses during cue- and stress-induced reinstatement tests of alcohol-seeking. In addition to the facilitatory effect of estrogen on the HPA axis, these sex differences could be due, in part, to impaired glucocorticoid receptor negative feedback and increased CRF Subscript 1 receptor signaling in female rodents.

Both glucocorticoid receptors and CRF Subscript 1 receptors are being pursued as potential targets for treatment of AUD, but most preclinical and clinical data examining medications that target these receptor systems have used male subjects. The few clinical studies that included female subjects were underpowered to examine for sex effects. In the single study conducted with females—who had anxiety and AUD—the CRF Subscript 1 receptor antagonist verucerfont reduced HPA responsivity without altering measures of alcohol craving.^91^ Considering the preclinical data indicating that CRF Subscript 1 receptor antagonists effectively reduce escalation in alcohol drinking in dependent male rodents, it is not known whether verucerfont would reduce measures of alcohol drinking in females with AUD.

Regarding glucocorticoid receptor antagonists, the mixed glucocorticoid receptor and progesterone receptor antagonist mifepristone (also known as RU-486) significantly reduced measures of alcohol craving and alcohol consumption in participants with AUD.^5^ These participants were predominantly male (the mifepristone treatment group was 82% male). Because of its progesterone receptor antagonism, mifepristone is used in females to terminate pregnancy. Thus, use of mifepristone in females may be confounded by its mixed pharmacological properties, with the progesterone receptor antagonism producing more serious side effects in females versus males.

More selective glucocorticoid receptor antagonists, such as CORT113176, are being pursued, but data for females are not available. Preliminary data in mice selectively bred for a high binge drinking phenotype determined that CORT113176 significantly decreased binge drinking in both male and female mice, and that female mice were more sensitive to the effect.^107^

Pharmacological strategies targeting the CRF Subscript 1 receptor and glucocorticoid receptor systems may be differentially effective in males versus females, and new strategies targeting these systems could have greater specificity for females.^92^ For example, inhibiting molecules that facilitate the transport of glucocorticoid receptors to their classical intracellular receptor might normalize high glucocorticoid levels in females. Likewise, compounds that target the CRF Subscript 1 receptor and shift signaling away from pathways that enhance CRF Subscript 1 receptor signaling might make females more resilient to stress-induced hyperarousal.^92^

Strategies targeting GABA Subscript A receptor–active neurosteroids or their biosynthesis may represent an approach to effectively treat AUD in males and females. Results from preclinical models suggest that chronic alcohol drinking or the induction of dependence in females does not significantly alter allopregnanolone levels, as is seen in males. These results are consistent with the idea that the ability of females to maintain endogenous levels of a GABAergic neurosteroid following chronic alcohol exposure may contribute to the reduced severity of their alcohol withdrawal phenotype. Alternately, strategies to enhance neurosteroid synthesis may exert a protective effect against further alcohol drinking in females, as has been proposed for males.^99^

Neurosteroid analogs with a longer half-life than allopregnanolone show promise as another effective strategy. For instance, brexanolone was recently approved for the treatment of postpartum depression. Currently, ganaxolone also is in clinical trials for treatment of postpartum depression, as well as for treatment-resistant depression, PTSD, and epilepsy. Preclinical results indicate that ganaxolone significantly reduces alcohol drinking in male and female mice (Figure 5, DA Finn and MM Ford, unpublished data, April 2013). Thus, neurosteroid analogs may be effective at reducing alcohol drinking in individuals with co-occurring AUD and depression or co-occurring AUD and PTSD, or in individuals with AUD who drink to alleviate stress and negative affect.

Finally, use of progesterone as a “prodrug” to increase allopregnanolone levels has been an effective strategy to decrease cue-induced craving and cortisol responses in small cohorts of male and female patients with co-occurring AUD and cocaine use disorder.^105^,^106^ The greatest reduction in craving was observed in male and female participants who had the highest allopregnanolone levels after progesterone administration.^105^,^106^ Thus, strategies to use allopregnanolone analogs with longer half-lives, or to stabilize or enhance levels of GABA Subscript A receptor–active neurosteroids such as allopregnanolone, may represent new efficacious treatments for both males and females with AUD.

Collectively, the importance of arriving at a more complete understanding of the neuroendocrine mechanisms underlying sex differences is clear, as treatment strategies and their effectiveness may revolve around sex differences in the endogenous steroid and neurosteroid environments and in sexually divergent downstream signaling mechanisms. In addition, variations in neurosteroid physiology also may help explain individual differences in susceptibility to AUD, vulnerability to relapse, and the negative health consequences of alcohol intake.
